# Osteomyelitis by *Microsporum canis* and *Staphylococcus* spp. in cat (*Felis catus*) – case report

**DOI:** 10.1186/s12917-024-03904-4

**Published:** 2024-02-19

**Authors:** Fernanda O. Soares, Isabel R. Rosado, Marcus V. C. Sousa, Carolyne F. Dumont, Joely F. F. Bittar, Ian Martin, Marcelo E. Beletti, Roberta T. Melo, Endrigo G. L. Alves

**Affiliations:** 1 Hospital Veterinário Uniube, Universidade de Uberaba, Uberaba, Minas Gerais Brazil; 2 Laboratório de Patologia Animal - PAVET, Universidade de Uberaba, Uberaba, Minas Gerais Brazil; 3 Laboratório de Epidemiologia Molecular - LEPIMOL, Universidade Federal de Uberlândia, Uberlândia, Minas Gerais Brazil; 4 Programa de Pós-Graduação em Ciências Veterinárias, Universidade Federal de Uberlândia, Av. Ceará, s/n, Bloco 2D43, Uberlândia, CC 38402-018 Brazil

**Keywords:** Domestic cat, Mandible, Microbial sensitivity test, Osteomyelitis

## Abstract

**Background:**

*Staphylococcus spp* and *Microsporum canis* are zoonotic microorganisms which can cause infections and systemic diseases. The bone infection is usually caused by invasion of pathogen through the hematologic route. Mixed osteomyelitis caused by bacteria and fungi is rare, and to date, there have been no reports of mixed osteomyelitis with *Staphylococcus* spp. and *Microsporum canis*.

**Case presentation:**

This essay reports an atypical presentation of mixed osteomyelitis (*Staphylococcus* spp. and *Microsporum canis*) in a domestic cat. A 15-month-old female Persian cat was presented to a veterinary service; the main complaint was the appearance of a nodule in the mandibular ventral rostral region. A radiographic exam performed on the animal showed proliferative and osteolytic bone lesions. The patient was submitted to a biopsy for histopathological evaluation, along with bacterial and fungal cultures. Results showed mixed osteomyelitis by *Staphylococcus* spp. and *Microsporum canis*. Microbial Sensitivity Test was performed to choose a more suitable treatment. Two surgical procedures were executed to resect and curette the lesion, and treatments with anti-inflammatory, antibiotic, and antifungal drugs were established, showing a positive clinical evolution. After 8 months of treatment, the patient’s owner moved to a different city, and the animal was seen by other veterinarians, who followed along with the same treatment. However, due to complications and a diminishing quality of life over 4 years of diagnosis, the patient was euthanized.

**Conclusion:**

Given the above, mixed osteomyelitis is difficult to treat and can cause losses of life quality resulting death, especially in infections where *M. canis* is the agent causing the disease. Bacterial osteomyelitis is more frequently reported. But the lack of investigation of microorganisms other than bacteria, such as fungal cases, may imply in underdiagnosed cases. Treatment of osteomyelitis can be difficult considering the difficulties in isolating the pathological agent, resistance to the drug used, prolonged treatment time, and cost.

## Background

Osteomyelitis is an inflammation that can affect all bone components and it is usually associated with systemic bacterial infections or caused by metallic bone implants failure that can be colonized by bacterial, forming biofilms [1, 2]. However, it can also be of fungal or viral etiology [3]. It is a severe bone lesion, with chronic aspect, requiring treatment and can threaten the patient’s life [4, 5]. The lesions start small and evolve gradually over time if not treated [5], with a deforming appearance, potentially causing necrosis, resorption, and neoformations [1, 6]. 

Fungal osteomyelitis are rare and usually occurs systemically in a non-suppurative multifocal way, producing osteolysis [5–7]. The most prevalents cases are caused by *Histoplasma capsulatum, Coccidioides, Blastomyces* and *Cryptococcus* (Table 1). Others species can be affected for *Microsporum canis* although not restricted to osteomyelitis, atypical cases are often reported in dermatitis in humans. Major risk factors associated with bone infection include the extent of injuryand devitalization of adjacent soft tissues, severe bone damage, treatment delay, immunosuppression, comorbidities, and prolonged maintenance of central venous accesses [5, 6, 8, 9]. In felines, mandibular osteomyelitis is as common as neoplastic formations and is frequently associated with periodontal diseases originating from dental fractures infected by the oral microbiota [10–13]. . However, cases of osteomyelitis caused by *Microsporum canis* have never been reported. Nevertheless, we know that in this species, dermatitis caused by this fungus is common, and this could be a possible entry point. It is noteworthy that this condition is a zoonosis, with a feline with fungal dermatitis being a reservoir for humans [14].

**Table 1:** Non cutaneous presentations and osteomyelitis infections caused by fungi in cats, dogs and humans

Ref	Total patients	Specie	M/F	Age (mean)	Disease course (mean)	Region/lesion	Treatment method	Agent
[9]	1	Cat	M	4-month	6 months	Extensive lytic lesion involving the distal metaphysis of the right femur	5 weeks of high-dose itraconazole therapy	Unknown
[32]	7	Cat	M and F	8 months to 8 years	3 to 12 weeks	Bony involvement, including lameness, soft tissue syelling of limbs or joints, and pain on bone palpation.	No treatment	*Histoplasma capsulatum*
[33]	18 of 101	Cat	49 M and 52 F	Median age 6.5	6 months	*Histoplasma capsulatum* was confirmed in one organ (*n* = 83) or more than one organ (*n* = 18) via multi-needle aspiration and cytopathology of the lung (*n* = 18), liver (*n* = 17), spleen (*n* = 17), lymph node (*n* = 17), painful skin lesion (*n* = 5), bone (*n* = 2), oral lesion (*n* = 2) and one of each eye, kidney and unknown abdominal mass. 18 of 101 presented lameness.	Itraconazole followed by fluconazole	*Histoplasma capsulatum*
[34]	3 of 51	Cat	30 NM and 21 F	1 to 15.5 years (mean ± SD 6.8 ± 4).		Lytic/proliferative bone lesion (*n* = 3/51 [0,17%]). Specifically in a male neutered cat with soft tissue swelling was identified, with several months’ history of lameness.	Oral fluconazole and Itraconazole	*Coccidioides immitis* and *Coccidioides posadasii*
[35]	2	Dog	M	8	2 months	A 5-cm-diameter subcutaneous nodule on the ventral neck, enlarged with sinuses draining a purulent discharge.	At first treatment, was instituited 10 days of therapy with cephalexin (25 mg/kg twice daily), no improvement was observed. Before diagnostic of the *M. canis*, the therapy consisted of itraconazole use (10 mg mL-1 once daily, for unknow period) and surgical excision of the nodule.	*Miscrosporum canis*
[35]			F	4	Acute	A 3-cm-diameter, subcutaneous nodule on the groin, close to the mammary gland (pseudomycetoma). Another nodule of < 2 cm in diameter, developed in the axilla after one month of therapy with itraconazole.	3 months of itraconazole therapy (10 mg/kg once daily)	*Miscrosporum canis*
[24]	1	Human	M	45		Left tibial osteomyelitis, accompanied by skin fungal infection of the ipsilateral heel. The patient had a history of injury from a rusty object, which penetrated the anterior skin of the left tibia middle segment causing subsequent bone infection.	Surgical debridement and vacuum sealing drainage (VSD) three times followed by antimicrobial therapy sequentially with ceftizoxime (30 days) and piperacillin zobartan (35 days) and potassium permanganate tablets for dermatophytosis that lasted 3 days.	*Staphylococcus aureus*, Corynebacterium and dermatophytosis fungus caused by an unidentified species
[2]	1	Human	M	52		Osteomyelitis of the calcaneus, initially presented with a pilon fracture requiring temporary external fixation while awaiting definitive fixation.	Surgical debridement and 3 months of itraconazole.	*Trichophyton rubrum*
[36]	1	Human	F	32		A cluster of papules that ranged in size from 0.5 to 1.0 cm on the knee, with a mycetoma-like.	Oral fluconazole treatment was initiated (200 mg/day)	*Miscrosporum canis*

NM = neutered males; M = males; NF = neutered females; F = females

## Case presentation

The present case report is about a spayed 15-month-old female domestic Persian cat, weighing 3.1 kg. The owner brought the animal to a veterinary clinic in Uberaba – MG and the main complaint was a progressively growing nodule, for long of eight months, in the mandibular ventral rostral region. The owner reported a good general state, the animal had no access outside of the residence it lived at and had contact with another healthy cat from the same household. Denies fights between the animals, traumas, or other situations that could be related to the affected area. The patient had a history of intense dermatitis., which resulted in grave alopecia in the whole body when the animal was three months old; on this occasion, the animal was treated for dermatophytosis with antifungal medication and recovered completely. When the patient was around one year old, the owner noticed a growth in the mandibular region and performed a needle puncture in the area, with unknown asepsis, thinking it was an abscess.

**Fig. 1:**
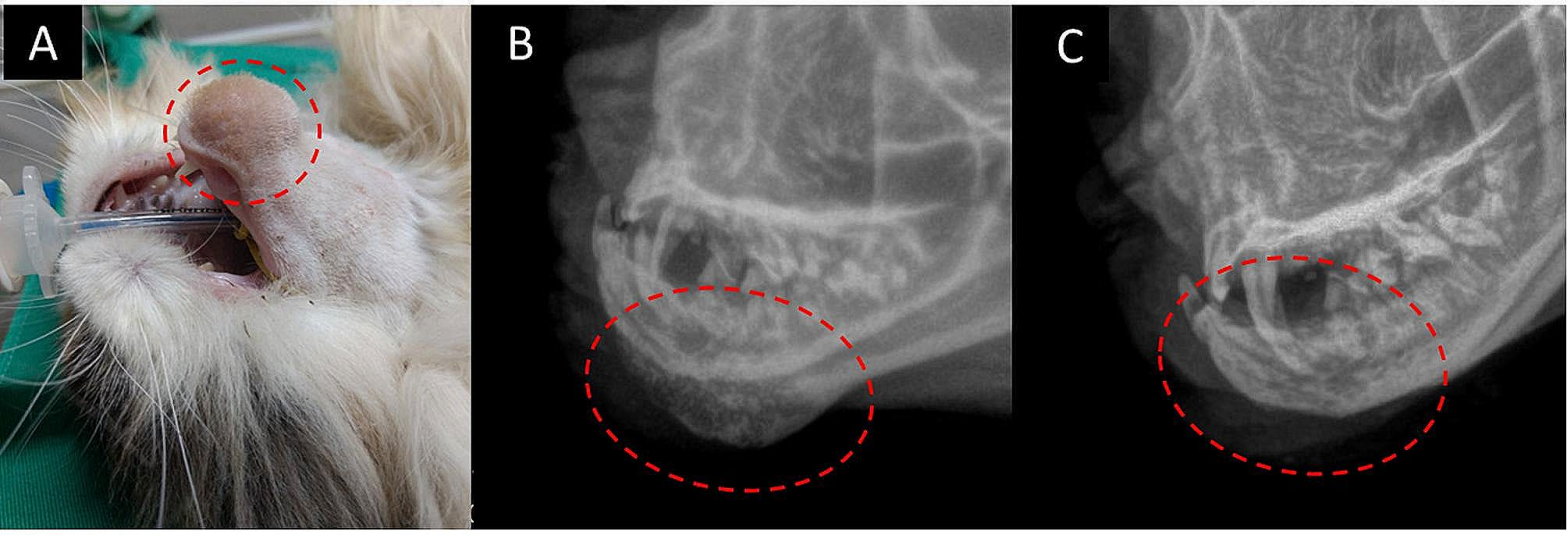
15-month-old female Persian cat with mixed osteomyelitis (Microsporum canis and Staphylococcus spp.) showing a circumscribed non-ulcerated growth in the mentonian region of the mandible (**A**). Skull radiographic image in a left oblique projection of a cat with suspected osteomyelitis or neoplasia. The red dotted area shows a volume increase with proliferation areas and areas of osteolysis (**B**). Skull radiographic image in left oblique projection after 120 days of treatment. The red dotted area shows a reduction in volume and improvement in bone appearance (**C**)

At the general clinical exam, the animal did not present any other abnormalities. As for the increase in volume in the mandibular ventral-rostral region, it presented a firm consistency, non-ulcerated, normal local temperature, no painful sensitivity to palpation, and no changes in the coat (Fig. 1 - A).

In the blood count exam, the red series showed an increase in RDW (Red cell Distribution Width) without anemia. In the white series, it is possible to observe a slight increase in immature neutrophils and monocytes without leukocytosis (Table 2). Radiographic examination revealed a periosteal reaction with a smooth and uniform margin, lost of normal trabecular pattern with proliferation and bone remodeling and radiolucent areas of millimeter dimension and destructive character in the mentonian region of the left hemi-mandible (Fig. 1 - B), suggestive of osteomyelitis or bone neoplasia.

**Table 2:** Results of hemogram and biochemical series

	Pre-treatment	After 120 days of treatment	
Blood count			Reference values
Red blood cells in millions	9,93	8,12	5,5–8,5 u^3^
Hemoglobin	13,90	13,10	12,0–18,0 g/dL
Hematocrit	43,60	38,70	37,0–55,0%
MCV	43,90	47,66	60,0–77,0 fL
MCH	13,99	16,13	19,0–23,0 pg
MCHC	31,88	33,85	31,0–33,0 g/dL
RDW	15,2	17,4	13,5–15,0%
**Leucogram**			
Leukocytes	15.300	6.700	6.000–17.000/mm^3^
Rods	306	134	0–300/mm^3^
Segmented	6.120	3.752	3.000–11.000/mm^3^
Eosinophils	459	268	100–1.250/mm^3^
Lymphocytes	7.497	2.345	1.000–4.800/mm^3^
Monocytes	918	201	150–1.350/mm^3^
Platelets	411.000	316.000	200.000–900.000
Biochemistry			
Oxalacetic transaminase	28,0	27,0	26,0–43,0 U/L
Alkaline phosphatase	15	13	< 50 U/L
**Bilirubin**			
Total bilirubin	0,15	0,17	0–0,4 mg/dL
Indirect bilirubin	0,05	0,08	0–0,1 mg/dL
Direct bilirubin	0,06	0,09	0–0,3 mg/dL
**Protein**			
Total proteins	7,1	6,49	5,8–8,5 g/dl
Albumin	3,5	3,11	1,9–3,80 g/dL
Globulins	3,6	3,38	2,3–5,0 g/dL
Albumin/globulin ratio	0,97	0,92	0,5–1,1 g/dL
**Lipidogram**			
Total cholesterol	123	179	95–130 mg/dL
HDL cholesterol	43	63	42–58 mg/dL
LDL cholesterol	73,2	106,2	69–94,9 mg/dL
VLDL cholesterol	6,8	9,8	6–8 mg/dL
Triglycerides	52	49	50–100 mg/dL

**Fig. 2:**
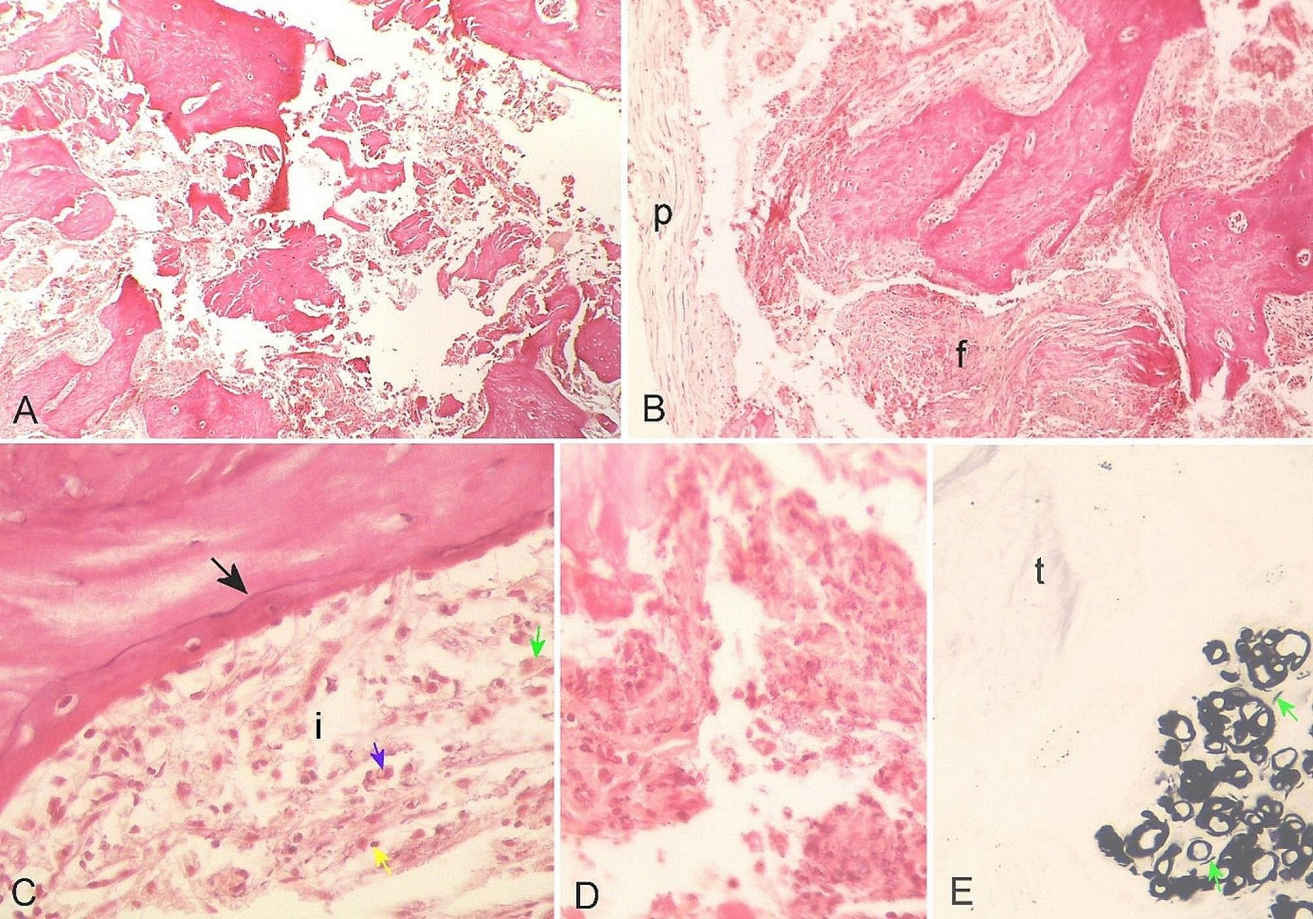
Mandible bone tissue photomicroscopy of a cat with mixed osteomyelitis. (**A**) Panoramic view where loss of bone structure is observed. H&E. (**B**) Periosteum (p) detached from the bone matrix and area of fibrosis (f). H&E. (**C**) Basophilic cementing line (black arrow) demarcating the transition between old and newly formed bone tissue bordered by an inflamed area (i), where lymphocytes (yellow arrow), plasma cells (blue arrow) and foamy macrophages (green arrow) can be seen. H&E. (**D**) Area of fibrosis with degenerated neutrophils. H&E. (**E**) Black-colored structures of rounded and filamentous (green arrow) morphology adjacent to scarcely stained fibrous tissue (t). Grocott technique

A biopsy of the lesion was performed for histopathological examination and for microbiological cultures. For the histopathological evaluation, a Hematoxylin and Eosin stain was made (H&E) [15], along with Gomori’s methenamine silver impregnation method for the histopathological visualization of structures [16]. For the H&E method, losses of bone tissue architecture were observed, characterized by disaggregation/degeneration of bone matrix (Fig. 2 - A) and a thinned periosteum, partially detached from cortical bone (Fig. 2 - B), which can be a processing artifact or not. A thin basophilic cementation line marks the transition between the old bone matrix with empty lacunae and the new bone matrix with lacunae filled with osteocytes, evidencing neo-osteogenesis. (Fig. 2 - C). There are different foci of inflammation surrounding the bone matrix, marked by fibrosis, and chronic inflammation, mediated by lymphocytes, plasma cells, neutrophil degenerating cells, and foamy macrophages (Fig. 2 - Ci). Outlines of hemorrhagic necrosis, traces of fibrosis, and moderate aggregates of degenerated neutrophils, free of malignancy, were also identified (Fig. 2 – D). Based on the histopathological findings, chronic osteomyelitis was diagnosed. Discrete structures of capsulated round-filamentous, peripheral to the bone trabeculae, and a discretely refraction morphology were noticed, compatible with a fungal structure, measuring 15,8 ± 3,9 μm, which was confirmed with Gomori’s stain method (Fig. 2 - E).

In the same time, the sample of bone fragment sent for biopsy was subjected to culture, there was evidence of growth and isolation of *Microsporum canis* and *Staphylococcus* spp. The *Microsporum canis* isolate on Sabouraud dextrose agar - SDA (Merck®), cultures were incubated for seven days, at 20–25 °C in the dark [17]. For *Staphylococcus* spp., it was isolated and identified according to the British Standards Institution, the culture was inoculated on Blood Agar supplemented with 5% lysed horse blood, once grown colonies were identified by Gram coloration. Gram positive colonies were submitted to the Catalase test (Laborclin®), tested for coagulase test (Laborclin®) and Mannitol Agar (Merk®) [18]. 

An antimicrobial susceptibility test [19] was carried out in triplicate, which revealed that the bacteria were sensitive to azithromycin, amoxicillin with clavulanic acid, and cephalexin and resistant to the following antibiotics: vancomycin, clindamycin, and neomycin, also showing intermediate resistance to erythromycin, which allowed classifying the pathogen as multidrug-resistant (MDR) [20]. Sensitivity was found in the antifungigram to all antifungals evaluated, including ketoconazole, miconazole, clotrimazole, fluconazole, itraconazole, and voriconazole. The interpretation of the inhibition halos diameters happened according to the interpretation criteria recommended by the Clinical and Laboratory Standards Institute (CLSI) 2020 [19]. 

Following the mixed osteomyelitis diagnosis, the animal was treated with meloxicam 0.03 mg/kg, q24h for 10 days, itraconazole 10 mg/kg, q24h for 120 days, and azithromycin 10 mg/kg, q24h for 3 days, being repeated every 10 days until 120 days. The plan was to carry out monthly follow-ups, however, the owner only returned with the animal 8 months after the treatment had started. At that time, new hematological tests were performed (complete blood count, aspartate aminotransferase, alkaline phosphatase, bilirubin, and total proteins, along with their fractions), and the results obtained did not show abnormalities. In addition, a new radiographic examination of the mandible was also performed, which revealed discrete bone modifications, with reduction in volume and improvement in bone appearance (Fig. 1 - C), suggesting a non-complete remission of the signs.

After that period, the follow-ups were done by other veterinarians. The nodule started growing again, and a new surgery was performed to remove it 14 months after the first intervention. The surgery was able to keep the patient comfortable for a few months. The mass continued growing despite the drug treatment, causing skin ulcers, feeding problems, and a significant loss in quality of life.

## Discussion and conclusions

Fungal and bacterial osteomyelitis can be acquired by foreign bodies, open wounds, or hematogenous by inhaling spores [5–7, 9, 18]. Most bone infections involve the metaphysis, flat bones, vertebral bodies, or vertebral discs [7]. The bone in this patient was affected at the height of the rostral mental foramen and the mandibular symphysis which is in line with literature data [7, 21]. The patient had a history of a puncture in the mandible region without previous antisepsis, which may have inoculated cutaneous microorganisms into the bone affected according with literature by foreign bodies. Another hypothesis raised is that the microorganisms may have been inoculated into the bone by a small wound not noticed by the owner. However, the possibility of hematogenous infection cannot be ruled out.

*Coccidioides immitis*, *Blastomyces dermatitidis*, *Histoplasma capsulatum* and *Cryptococcus neoformans* are rare microorganisms described in the literature isolated in fungal osteomyelitis [3, 8]. *Aspergillus* spp. is the most frequently reported cause of osteomyelitis and anthritis in veterinary medicine [1, 22]. The pathogen isolated restricted to the mandible, *Microsporum canis*, had not yet been reported as an agent of bone infection of cat and any other species. Dermatophytes are microorganisms that possess a dermatophytic metabolism unique to the keratin in which they secrete sulfite, as well as keratinases, in order to carry out keratinolysis [23]. It is a dermatophyte fungus that can invade the stratum corneum of the epidermis and the keratinized tissues derived from it, such as the skin, nails, and hair of humans and animals [3]. However, in the present case there were no signs of dermatitis.

Immunological deficiencies, the number of microorganisms inoculated, the strain’s virulence, traumas associated with tissue ischemia, and the presence of a foreign body inside the tissue are some of the factors that can boost the disease [5, 6]. Although osteomyelitis is rarely caused by dermatophyte, Kong et al. (2022) reports a case in which a 45-year-old man suffered a puncture wound from a sharps object [24]. The lesion caused mixed osteomyelitis with fungal, *Staphylococcus aureus* (*S. aureus*) and *Corynebacterium* involvement. Similarly, the patient in the case we report had a history of generalized fungal dermatitis when younger, which leads to the assumption that this infection had installed itself on the bone at that time and evolved slowly until the increase in the size of the area was noticed. *Microsporum canis* is an opportunistic pathogen and can penetrate the skin causing invasive infections [25, 26]. However, the hypothesis of a more virulent strain that used unknown mechanisms to invade the tissue and surpass the host’s immune system cannot be deserted. Infiltration of *M. canis* can also occur through scratching and invasive procedures, or through maintenance in hair follicles or intrafollicular hairs for maintenance and access to a nutrient source, in cats and humans [27]. The development of atypical forms in cats is associated with pseudomycetoma formation with the possibility of infiltration and systemic dissemination [28].

The leukocyte alterations are compatible with a bacterial infectious process, with a slight increase in rods and monocytes which is justified by the mixed infection. In bacterial osteomyelitis, the acute form of the disease is the one that can present with abnormalities in blood work. In some cases, neutrophilic leukocytosis and a left shift are observed, which indicates a bacterial infection. At the chronic stage of the disease, as in the patient described in this report, the blood count exam will rarely show important hematological abnormalities, corroborating with literature descriptions [4]. In fungal osteomyelitis, the hemogram is within normal ranges and morbidity in uncommon cases may cause changes such as nonregenerative anemia and leukocytosis, especially when the infection spreads [5, 7].

The radiographic appearance of a bone infection begins with an increase in soft tissue volume, with gradual structural changes not evident at first. It was possible to identify bone proliferation on the surface along with more internal osteolysis [5, 6]. Radiographic evaluation allowed assessment of the extent of the lesion, identifying active periosteal reactions and bone remodeling with destructive characteristics compatible to bone cancer, and osteomyelitis.

Possible differential diagnoses include primary bone neoplasms, metastases, lymphoid tissue neoplasms, bacterial osteomyelitis, and abscesses [5, 7]. For imaging findings that resemble those found in osteosarcomas, it was necessary to perform a cytological examination or histopathological biopsies to confirm the diagnosis [2]. 

Combined with histopathological findings, the growth of *M. canis* and *Staphylococcus* spp. in fungal and bacterial cultures confirmed the diagnosis of osteomyelitis in the present case. Microbiological culture is the primary test for diagnosing bacterial and mycotic osteomyelitis. After isolation of the microorganism, it is possible to perform antimicrobial susceptibility testing and establish an effective therapeutic protocol [7]. 

Itraconazole was chosen as an antifungal drug considering it is a broad-spectrum drug and has fewer side effects compared to other antifungals. Fungal osteomyelitis treatments are time-consuming and expensive. The average use of drugs will vary according to the signs observed in each case, but it is always necessary to use the drugs up to one month after the clinical resolution of the signs. The medications most used in cases of fungal infections include fluconazole, ketoconazole, amphotericin B, and itraconazole. Amphotericin B is more frequently used in human medicine and should be handled with care due to the nephrotoxic effects it can cause, especially in feline species [8].

Even with a careful choice and administration of drugs, relapses may occur, and in cases of refractory to treatment, amputations may be necessary [8]. Due to a non-complete remission of radiographic signs during a long period of treatment, it was decided to switch from itraconazole to fluconazole. The use of azithromycin when treating infections caused by *Staphylococcus* spp. is due to the sensitivity of the agent to the drug and as a result of some facilities related to the dosage in feline patients.

In chronic osteomyelitis, sclerosis, necrosis, and reduced vascularization occur, limiting the ability of the immune system to eliminate the infection and reducing the penetration of drugs into the infection focus area [1, 13]. Prolonged use of high-dose antimicrobials increases the risk of liver and kidney disorders, which may lead to a poor prognosis [29]. Despite the use of effective antimicrobials against the pathogens isolated in the present report, the difficulty of penetration of the drugs in the affected region may have been an important factor in the non-complete remission of the disease. The low response to treatment can also be attributed to the development of resistance, such as overexpression of membrane transporters and altered ergosterol biosynthesis, to itraconazole and fluoconazole, both triazoles [30]. The ended up progressing to loss of quality of life, resulting in euthanasia.

Bacterial osteomyelitis are more commonly reported than ones of mycotic, protozoal, or viral etiology. The lack of description of osteomyelitis by microorganisms other than bacteria is related to the non-investigation of these agents, which may imply these cases are underdiagnosed [31]. In a general context, osteomyelitis of any etiology is demanding to treat due to difficulties in isolating the pathological agent, misuse of proven multi-resistant drugs, prolonged time of treatment, and onerousness. Fungal osteomyelitis is rare. *Microsporum canis* is a fungus commonly described in skin diseases and has the potential to cause osteomyelitis.

## Data Availability

All data supporting the findings of this study are available within the paper and its Supplementary Information. The radiographic images are provided in Fig. 1 A, B and C, as well as the images and description of the histologic slides used in this study, Fig. 2 A to E. The microbiological analysis report presenting the diagnosis of *M. canis*, which support the findings of this study are available from the authors, but there are restrictions on the availability of these data, which were used under license from the Laboratory Tecnologia em Sanidade Animal (TECSA) for the current study and are therefore not publicly available. The data are, however, made available by the authors upon reasonable request and with permission from the laboratory TECSA.

## Abbreviations

CLSI: Clinical and Laboratory Standards Institute
RDW: Red cell Distribution Width
H&E: Hematoxylin and Eosin
SDA: Sabouraud dextrose agar
MDR: multidrug-resistant
